# Availability and affordability of anticancer medicines at cancer treating hospitals in Rwanda

**DOI:** 10.1186/s12913-023-09706-y

**Published:** 2023-06-30

**Authors:** Clement Rurangwa, Jerome Ndayisenga, Jurdas Sezirahiga, Eric Nyirimigabo

**Affiliations:** 1 Immunization, Vaccines and Health Supply Chain Management, College of Medicines and Health Sciences, EAC Regional Centre of Excellence for Vaccines, University of Rwanda, Kigali, Rwanda; 2 African Research and Community Health Initiative (ARCH Initiative), Kigali, Rwanda; 3 Rwanda Food and Drug Authority (Rwanda FDA), Kigali, Rwanda

**Keywords:** Availability, Affordability, Anti-cancer medicines, Cancer treating hospitals, Rwanda

## Abstract

**Background:**

Availability and accessibility of anti-cancer medicines is the pillar of cancer management, and it is one of the main concerns in low-income countries including Rwanda. The objective of this study was to assess the availability and affordability of anticancer medicines at cancer-treating hospitals in Rwanda.

**Methodology:**

A descriptive cross-sectional study was conducted at 5 cancer-treating hospitals in Rwanda. Quantitative data were collected from stock cards and software that manage medicines and included the availability of anti-cancer medicines at the time of data collection, their stock status within the last two years, and the selling price.

**Results:**

The study found the availability of anti-cancer medicines at 41% in public hospitals at the time of data collection, and 45% within the last two years. We found the availability of anti-cancer medicines at 45% in private hospitals at the time of data collection, and 61% within the last two years. 80% of anti-cancer medicines in private hospitals were unaffordable while 20% were affordable. The public hospital that had most of the anti-cancer medicines in the public sector provided free services to the patients, and no cost was applied to the anti-cancer medicines.

**Conclusion:**

The availability of anti-cancer medicines in cancer-treating hospitals is low in Rwanda, and most of them are unaffordable. There is a need to design strategies that can increase the availability and affordability of anti-cancer medicines, for the patients to get recommended cancer treatment options.

**Supplementary Information:**

The online version contains supplementary material available at 10.1186/s12913-023-09706-y.

## Introduction

Cancer is considered to be a worldwide problem and is among the primary root causes of death [1]. Approximately 9.9 million deaths and 19.3 million new cases were reported in 2020 around the world, with most deaths and cases coming from low and middle-income countries (LMICs) when compared to High Income countries (HICs). In Africa, 711,429 deaths and 1.1 million new cases were reported, as well as 8835 new cases and 6044 deaths in Rwanda in 2020 [2, 3].

Access to chemotherapy has a significant impact in the control of cancers, but high cost of anti-cancer medicines challenge availability [4, 5]. High cost of anti-cancer medicines is a global issue, and places financial burden to patients, families and societies in both LMICs and HICs; however, economic factors including wealth levels determine the pattern of affordability. Individuals diagnosed with cancer are likely to declare bankruptcy and experience other financial hardship than individuals with no cancer [6–8]. While most generic anti-cancer medicines are affordable to government purchase in Sub-Sahara African countries including Rwanda, they are unaffordable to individual purchase [3].

A technical report from WHO mentioned that the availability of anti-cancer drugs is 32.0% in lower-middle-income and 57.7% in low—income countries [9]. Africa has a small number of publications on the availability and affordability of anti-cancer medicines, and studies done reported a scarcity of important medicines including anti-cancer medicines [4, 10]. In LMICs, much of the population has limited access to medicines, either because of low availability rate or because patients must pay for the medicines. This in turn pushes them into deprivation, poverty or premature death [4].

Promoting access to anti-cancer medicines found on the WHO Model List of Essential Medicines (EML) can improve affordability of high value medicines to individual purchase. The WHO EML helps countries in selecting effective, safe, and cost-effective medicines to develop National Essential Medicines Lists [11, 12]. 41.0% of WHO EML for treatment of cancers are aligned with Rwanda National Essential Medicines for Treatment of cancers [3]. essential medicines for treatment of cancer in Rwanda are suggested by the Ministry of Health, and are expected to be available to cancer treating hospitals [13].

Absence of insurance coverage reduces access to anti-cancer medicines in patients living with cancer and increases financial burden [14]. High levels of poverty in LMICs including Rwanda reduces access to anti-cancer medicines. In Rwanda, a community-based health insurance scheme is a form of Universal Health Coverage that covers about 90% of the population, and patients pay 10% of medical bills. It enables access to some oncology treatments like surgery and radiotherapy, but chemotherapy is uncovered [15–17]. However, the Ministry of Health is making advocacy to integrate chemotherapy into community-based health insurance scheme treatment [18].

Studies showed that anti-cancer medicines are expensive to individual purchase, and it was proven that unaffordability of anticancer medicines leads to treatment abandonment [3, 4, 19]. Improved treatment, prevention, and early detection helps to lower death mortality while poor availability and affordability lead to a negative impact on quality of life of the cancer suffering patients [20]. To reduce mortality from common curable cancers in Rwanda, Rwanda Biomedical Center introduced a National Cancer Control Plan (NCCP 2020–2024), and the strategies include establishment of systematic therapy capacity at university teaching hospitals and promoting equitable access to newer cancer treatments [18].

Rwanda promoted availability and affordability of anti-cancer medicines by developing partnerships with philanthropic organizations [3]. A robust cancer disease control started in 2012 with the establishment of the BUTARO Cancer Center of Excellence (BCCOE). Ever since, a lot has been attained in terms of cancer control plan, advocacy, medical diagnosis, therapy, and screening [17]. The improved and equitable treatment is available in the country as BCCOE provides anti-cancer medicines free of charge under Partners in Health support [18]. By 2020, BCCOE treated over 11, 116 cancer patients, and a high number of patients reported perception of receiving improved quality of cancer care [21, 22]. Localization of the center in rural areas of Northern province promoted equitable access to quality care; however, cancer care needs to be dispersed in other areas of the countries to improve geographical access [23].

Limited studies have been conducted to explore availability and affordability of anti-cancer medicines in Rwanda. Nael (2016) focused on cost while Nkurunziza (2021) focused on access and both studies emphasized on Pediatric Cancer [24, 25]. Another study of Kizub et al. (2022) reported access and affordability of WHO Essential Medicines for cancer in Sub-Saharan countries, but did not report availability in Rwanda while different factors affect accessibility [3]. Limited information on anti-cancer medicines has a high direct effect on cancer health care in Rwanda. Consequently, it hinders the adoption of effective measures the government and partners can use to ensure that cancer patients receive efficient and inexpensive anti-cancer medicine accordingly. The aim of this cross-sectional study was to conduct an assessment to determine the level of availability and affordability of the cancer treatment drugs in the cancer treatment hospitals in Rwanda.

## Materials and methods

### Study design

This was a descriptive cross-sectional study conducted to assess the availability and affordability of anticancer medicines at the cancer-treating Hospitals in Rwanda at the time of data collection in February 2022, and in the period of two years from August 2019 to August 2021.

### Study site

The study involved all five cancer treatment hospitals in Rwanda (Table 1). These included University Teaching Hospital of Kigali (CHUK), University Teaching Hospital of Butare (CHUB), King Faisal Hospital (KFH), Butaro Cancer Center of Excellence (BCCOE), and Rwanda Military Hospital (RMH).

**Table 1:** Locations, type and bed capacity of assessed hospitals

No	Name	Location	Type	Bed capacity
1	King Faisal Hospital (KFH)	Kigali city	Private	162
2	Butaro Cancer Center Of Excellence (BCCOE)	Northern province	Public	167
3	University Teaching Hospital of Kigali (CHUK)	Kigali city	Public	500
4	University Teaching Hospital of Butare (CHUB)	Southern province	Public	500
5	Kanombe Military Hospital (KMH)	Kigali city	Public	265

### Sampling technique

All cancer treating hospitals in Rwanda were selected in this study. We selected and reviewed all 33 anti-cancer medicines that were found on the list of essential medicines of Rwanda Ministry of Health for treatment of cancer in Rwanda [26].

### Data collection tool

Data were collected by using data collection form developed for this study. We did a pilot study to evaluate the effectiveness of data collection form. During pilot study, we surveyed one hospital (KFH) and recorded data regarding all 33 selected anti-cancer medicines. Pilot study provided satisfactory results for the data collection form to be used in our study.

### Data collection process

Data collection covered 2 weeks of February 2022. Our study assessed 33 anti-cancer medicines listed in the National List of Essential Medicines for Adults [13, 26].We recorded the data on availability and affordability for the day of visit and for the last two years. The medicine was said to be available within the last two years when there was evidence of being in stock between August 2019 and August 2021. To determine affordability within the last two years, we calculated the average selling price for every medicine within the study period. At each hospital, a staff member who is involved in stock management of anti-cancer medicines and/or pharmacy activities helped us to retrieve information from Stock cards, Software, Annual quantification report, prescribed anti-cancer medicines, lists of anti-cancer Medicines received, and Patients’ information Record books.

Data were collected from software that are used for stock management in selected hospitals (Electronic Logistics Management Information System), and stock cards that have information on anti-cancer medicines. Data were recorded on a pre-developed data collection tool for our study. Recorded data included availability of anti-cancer medicines at the time of data collection, its stock status within the last two years, and the selling price.

### Data analysis procedure

The availability of anti-cancer medicine was analyzed by comparing the stock available to the average monthly consumption for the study period (August 2019 up to August 2021). Anti-cancer medicines that were present in stock were classified as “Available” while medicines that were not present were classified as “Unavailable”. Stock levels that fall below quantity for one month of consummation were categorized as “Understock”, stock levels between one to two months of consummation were categorized as “Stock according to plan”, and stock levels that exceed two months of consummation were categorized as “Overstock”. Products with quantity equal to zero were categorized as “Stock out”. The quantitative data were analyzed by using Statistical Package for the Social Sciences. We analyzed the availability and affordability of the anti-cancer medicines by analyzing the cost and pricing of the medicine.

The affordability of anti-cancer medicines was assessed at cancer treating hospitals, which had at least one of surveyed anti-cancer medicines within the last two years. WHO and Health Action International suggested that a treatment course requiring more than 1 day’s wage is unaffordable [27]. The lowest estimated wage of unskilled workers in Rwanda is 1000 Rwandan Francs (RWF), and a similar amount has been used in recent studies [3, 28]. Affordability was determined for each selected anti-cancer medicine that was available at the day of visit and/or within the last two years. Affordability was calculated as the price of medicines for one course of treatment divided by 1000 (Lowest wage in RWF of unskilled worker in Rwanda). We defined one course of treatment as anti-cancer medication for 30 days, which is recommended by WHO and Health Action International for researches conducted on chronic conditions including cancer. Anti-cancer medicine was categorized as affordable if the patient can afford the medicine with a wage of one day.

## Results

### Availability of anti-cancer medicines at the day of visit

Anti-cancer medicines were available at two out of five cancer treatment hospitals at the day visit, of which one was public and the other private. The public hospitals had availability of surveyed anti-cancer medicines 14 (42%) while private hospitals had an availability of 15 (45%). No stock of anti-cancer medicines was available at the other three surveyed hospitals though they are cancer treating hospitals that are expected to have all of the assessed anti-cancer medicines.

Table 2 below shows the availability of assessed anti-cancer medicines on the day of visit: 4 (12%) were available in public hospitals only, 9 (27%) were available in private hospitals only, 11 (34%) were available in both public and private hospitals, and 9 (27%) were not available in any hospital.

**Table 2:** Availability of anti-cancer medicines at the day of visit

Stock status	Public Hospital			Private Hospital		
	n	N=	%	n	N	%
Available (Qty > 0)	14	33	42	15	33	45
Understock (AMC < 1)	9	14	64	3	15	20
Stock according to plan (1 < AMC > 2)	5	14	36	4	15	27
Overstock (AMC > 2)	0	14	0	8	15	53
Unavailable (AMC = 0)	19	33	57	18	33	55

AMC: Average Monthly consumption, N: number of anti-cancer medicines considered for category, n: number of anti-cancer medicines fall within category.

### Availability of anti-cancer medicines within the last two years

Stock status within the last two years showed that three cancer-treating hospitals had anti-cancer medicines. Public hospitals had availability of anti-cancer medicines of 15 (45%) within the last two years, including one item that was not available in the stock at the time of data collection (Methotrexate 50 mg injection). The private hospital had availability of anti-cancer medicines of 20 (61%) of surveyed medicines within the last two years, including five items that were not available in the stock at the time of data collection (Methotrexate 2.5 mg tab, Methotrexate 50 mg injection, Vincristine 1 mg/ml injection, Letrozole 2.5 mg tab, and Rituximab 100 mg injection). Within the last two years, all items that were found in public hospitals were available in BCCOE, but 2 (6%) of surveyed medicines were also available in another cancer-treating hospital. Two cancer-treating hospitals had no anti-cancer medicines for the last two years. Table 3 below presents the availability of anti-cancer medicines for last the two years.

**Table 3:** Availability of anti-cancer medicines last two years

Stock status	Public Hospital			Private Hospital		
	n	N=	%	n	N	%
Available (Qty > 0)	15	33	45	20	33	61
Understock (AMC < 1)	11	15	73	5	20	25
Stock according to plan (1 < AMC > 2)	4	15	27	7	20	35
Overstock (AMC > 2)	0	15	0	8	20	40
Unavailable (AMC = 0)	18	33	55	13	33	39

AMC: Average Monthly consumption, N: number of anti-cancer medicines considered for category, n: number of anti-cancer medicines fall within category.

### Affordability of anti-cancer medicines

The affordability of anti-cancer medicines was assessed at cancer treating hospitals, which had at least one of surveyed anti-cancer medicines within the last two years. Of the total anti-cancer medicines administered at private hospitals within the last two years, 16 (80%) were unaffordable while 4 (20%) were affordable to the patients. Affordable medicines were Methotrexate (2,5 mg), Tamoxifen citrate (20 mg), Letrozole (2.5 mg), and Mycophenolate (500 mg). All anti-cancer medicines administered at BCCOE were provided at no cost to the patients. The Table 4 below represents availability and affordability of anti-cancer medicines in cancer-treating hospitals within the last two years. CHUK had two anti-cancer medicines, Tamoxifen citrate Tab 20 mg that was affordable (requiring 0.65 working days), and Methotrexate Inj 50 mg that was unaffordable (Requiring 11.00 working days).

**Table 4:** Affordability of anti-cancer medicines within the last two years

			Affordability*			
No.	Medicine		BCCOE	Other Public hospitals (3)	private hospital (1)	
1	Folinic acid Tab 15 mg		Free	NA	NA	
2	Bleomycin inj 15 mg		Free	NA	21.60	
3	Cyclosphamide Tab 50 mg		NA	NA	NA	
4	Cyclophosphamide inj 500 mg		NA	NA	6.40	
5	Doxorubicine powder for inj 10 mg		NA	NA	NA	
6	Doxorubicine powder for inj 50 mg		Free	NA	7.84	
7	Methotrexate Tab 2 and 5 mg		Free	NA	0.10	
8	Methotrexate inj 50 mg		Free	11.00	2.12	
9	Vincristine inj 1 mg/ml		Free	NA	3.20	
10	Mercaptopurine Tab 50 mg		Free	NA	NA	
11	Hydroxycarbamide capsule 200 mg		NA	NA	NA	
12	Hydroxycarbamide capsule 1 gr		NA	NA	NA	
13	Tamoxifen citrate Tab 20 mg		Free	0.65	0.22	
14	Anastrozole Tab 1 mg		NA	NA	NA	
15	Letrozole Tab 2.5 mg		Free	NA	0.07	
16	Cisplatin inj 1 mg/ml		Free	NA	6.72	
17	Imatinib mesylate Tab 100 mg		Free	NA	NA	
18	Melphalan Tab 2 mg		Free	NA	NA	
19	Trastuzumab powder for inj 150 mg		NA	NA	704.00	
20	Zoledronate inj 5 mg/100 ml		NA	NA	8.32	
21	Oxaliplatin inj 5 mg/ ml		Free	NA	24.96	
22	Carbooplatin inj 100 mg/ml		NA	NA	3.45	
23	Paclitaxel powder for inj 6 mg/ml		NA	NA	40.48	
24	Ifosfomide Powder for inj 1000 mg		NA	NA	NA	
25	Fluorouracil(5-fu) inj 50 mg/ml		Free	NA	2.08	
26	Fluorouracil(5-fu) cream 0.5%		NA	NA	NA	
27	Fluorouracil(5-fu) cream 1.0%		NA	NA	NA	
28	Fluorouracil(5-fu) cream 5.0%		NA	NA	NA	
29	Irinotecan inj 200 mg/ml		NA	NA	57.60	
30	Docetaxel inj 20 mg/ml		NA	NA	38.40	
31	Rituximab powder for injection 100 mg		Free	NA	296.00	
32	Cyclosporine Tab 25 mg		NA	NA	1.60	
33	Mycophenolate Tab 500 mg		NA	NA	0.66	

Tab: tablet, inj: injectable, mg: milligram, ml: milliliter, %: percentage, NA: Not Available

*Number of working days required for lowest paid unskilled worker to pay for a 30 days course of treatment.

## Discussions

In Rwanda, the availability of essential anti-cancer medicines is low with a total absence in three major referral hospitals that treat cancers. Cancer treating referral hospitals should have all anti-cancer medicines found on the National Essential Medicines List to promote geographical access to high value and effective medicines. While anti-cancer medicines are not currently covered by community-based health insurance scheme, there is a promising effort of the Ministry of Health advocating for integration. Insurance coverage can further promote both availability and affordability of the anti-cancer medicines. Low availability of anti-cancer medicines in Rwanda can result in delayed or omitted chemotherapy, which is a risk factor for the worse survival and progression of cancer diseases to advanced stages [29].

BCCOE, a single public cancer treating hospital that has anti-cancer medicines is located in the Burera district of the northern province, at a high distance from Kigali city. Most patients are poor, but BCCOE offers free anti-cancer medicines, social support, and free meals. BCCOE receives support from the international community principally Non-Government Organization Partners In Health (PIH), which covers the cost of medicines and supplementary services [17, 29]. While the patients get anti-cancer medicines free of charge at BCCOE, limited geographical access to the people living in other provinces prevents health equity, as highlighted by the study on geographical access to cancer care centers in Rwanda [23]. It is essential to design interventions that will build the capacity of public referral hospitals for chemotherapy to save the lives of cancer patients.

Our findings of 42% availability of anti-cancer medicines in public hospitals are comparable to the study done in Tanzania that found the availability of 50% of surveyed anti-cancer medicines [30]. We found availability of anti-cancer medicines that are higher than availability reported in Ethiopia, which reported availability of 34.8% for the lowest priced generic drugs and 2.8% for the originator brand medications [31]. The findings that anti-cancer medicines were available in the private sector more than in the public sector agree with a study conducted in Pakistan that found the availability of 71.9% for originator brands and 20.0% for lowest priced generics in the private sector against 31.4% for originator brands and 11.7% for lowest priced generics in public health facilities [32]. A study conducted in Ghana also reported overall low availability of anti-cancer medicines, but higher in the private sector for lowest priced generics (13.08%) and originator brands (5.38%) than the public sector for lowest priced generics (10.55%) and originator brands 2.46%. Low availability of anti-cancer medicines in the public sector compared to the private sector can show Government underfunding for cancer treatment or poor inventory management at public hospitals that result in a suboptimal procurement and utilization of anti-cancer medicines.

We found that the most available anti-cancer medicines were unaffordable (80%) to the cancer patients when the payment was recommended for the patients to have medicines. Our study findings agree with a study conducted in Mexico, a middle-income country that found 86% of surveyed anti-cancer medicines to be unaffordable [19]; however affordability reported in our study was higher to the affordability reported in Ethiopia, where all anti-cancer medicines were unaffordable to both public and private hospitals except anastrazole that was affordable in public sector [31]. For the unaffordable drugs, cancer patients pay a cost that is more than the wage of one day to get medicines. It exaggerates poverty, which worsens the patient’s outcome. The Unaffordability of most anti-cancer medicines shall call for an effort to make cancer-related chemotherapy available and accessible in low and middle-income countries that have a high percentage of poor populations.

Studies that assessed NCDS drug products reported moderate to high availability of medicines against other conditions including cardiovascular diseases, diabetes and asthma in Rwanda. It was reported that more than 70% of essential NCDs medicines are available in health facilities in Rwanda with exception of warfarin and beta-blockers [33]. Anti-diabetes metformin was reported to be available in all health centers, while salbutamol inhaler was found in 94.1% of health centers. Cardiovascular drugs including ACE inhibitors and Alpha-blockers were found in all hospitals accessed [34]. Another study conducted in rural area of Rwanda reported that they were no stock-out of essential drugs for hypertension, diabetes and asthma at district hospital in 2018 [35].

The limitation of the current study was based on the fact that many cancer-treating hospitals were not having stock of anti-cancer medicines at the time of data collection, and information on affordability was not sufficient to make an inter-sector analysis of affordability of anti-cancer medicines for public and private hospitals. Future studies shall highlight the state of affordability of anti-cancer medicines in public and private hospitals that treat cancers in Rwanda.

## Conclusion and recommendation

Our study found low availability of anti-cancer medicines in Rwanda, which can result in poor management of patients living with cancer. Tertiary and university teaching hospitals that provide chemotherapy service were not having anti-cancer medicines at the time of data collection. Cancer treatment in public hospitals only depended on BCCOE, a cancer-treating hospital that is supported by Non-Government Organization Partners In Health. Unavailability of anti-cancer medicines found on the National Essential Medicines List in all cancer treating hospitals reduces geographical access. Availability of anti-cancer medicines in the private sector was higher than in the public sector, which can be caused by the Government’s underfunding for cancer treatment, or poor stock management in public hospitals causing suboptimal consumption of anti-cancer medicines. Increasing the knowledge of needed anti-cancer medicines and required quantity, coupled with strategies for bulk procurement and negation of price, will increase the availability and affordability of anti-cancer medicines in Rwanda. Integrating ant-cancer medicines into community-based health insurance scheme can increase availability and affordability.

We recommended the Ministry of Health (MoH) to deploy a sufficient budget to generate knowledge on the epidemiology of cancer in Rwanda, treatment options, and availability of required resources. They shall also promote a policy of incorporating anti-cancer medicines under the coverage of community-based health insurance for the patients to pay at convenient prices. All cancer treating hospitals that have the option of chemotherapy shall ensure the availability of anti-cancer medicines in stock.

## Electronic supplementary material

Below is the link to the electronic supplementary material.

Supplementary Material 1

## Data Availability

The dataset used in this study can be made available upon reasonable request to the corresponding author.
